# Antimicrobial Resistance Gene Detection and Plasmid Typing Among Multidrug Resistant Enterococci Isolated from Freshwater Environment

**DOI:** 10.3390/microorganisms8091338

**Published:** 2020-09-02

**Authors:** Sohyun Cho, John B. Barrett, Jonathan G. Frye, Charlene R. Jackson

**Affiliations:** Bacterial Epidemiology and Antimicrobial Resistance Research Unit, United States Department of Agriculture, Agricultural Research Service, U.S. National Poultry Research Center, Athens, GA 30605, USA; Sohyun.Cho@usda.gov (S.C.); bennybarrett09@gmail.com (J.B.B.); Jonathan.Frye@usda.gov (J.G.F.)

**Keywords:** *Enterococcus*, antimicrobial, resistance, plasmid, freshwater, environment

## Abstract

In this study, mechanisms of antimicrobial resistance (AR) as well as the abundance and diversity of plasmids were determined among multidrug resistant (MDR) enterococci from surface water in GA, USA. A total of 51 enterococci isolates were screened for the presence of 27 AR genes conferring resistance to ciprofloxacin, erythromycin, tylosin, kanamycin, streptomycin, lincomycin, Quinupristin/Dalfopristin (Q/D), and tetracycline. A plasmid classification system based on replication genes was used to detect 19 defined Gram-positive plasmid replicon families. Twelve genes were identified as conferring resistance to erythromycin and tylosin (*erm*(B) and *erm*(C)), kanamycin (*aph*(3′)-IIIa), streptomycin (*ant*(6)-Ia), lincomycin (*lnu*(B)), Q/D (*vat*(E)), ciprofloxacin (*qnr_E. faecalis_*), and tetracycline (*tet*(K), *tet*(L), *tet*(M), *tet*(O) and *tet*(S)). Twelve different *rep*-families were identified in two-thirds of the isolates. While AR genes commonly found in human and animals were detected in this study among environmental enterococci, resistance genes could not be determined for many of the isolates, which indicates that diverse AR mechanisms exist among enterococci, and the understanding of AR mechanisms for environmental enterococci is limited. Diverse *rep*-families were identified among the enterococci recovered from the aquatic environment, and these *rep*-families appear to be quite different from those recovered from other sources. This work expands knowledge of AR gene reservoirs and enterococcal plasmids across a wider range of environments.

## 1. Introduction

Enterococci are Gram-positive bacteria that are a part of the intestinal microbiota of warm-blooded animals. However, as opportunistic pathogens, they are among the major causes of nosocomial infections. *Enterococcus faecalis* and *E. faecium* account for most of the enterococcal infections in humans, but other species, including *E. avium*, *E. casseliflavus*, *E. durans*, *E. gallinarum*, *E. hirae*, *E. mundtii*, and *E. raffinosus,* have also caused human infections [1]. The treatment of enterococcal infections is often complicated by antimicrobial resistant enterococci, particularly those that are resistant to multiple drugs, and this is a major problem for public health worldwide as it results in limited therapeutic options [2]. Enterococci are intrinsically resistant to a broad range of antimicrobials, such as β-lactams and aminoglycosides, and are also adept at acquiring resistance to antimicrobial drugs and disseminating antimicrobial resistance (AR) determinants via horizontal gene transfer [3].

Antimicrobial resistant enterococci released into surface water through human and agricultural wastes are a public health concern, not only due to human exposure to these bacteria through water-related activities, but also due to potential dissemination of genes encoding AR to other bacteria present in the environment. However, studies reporting enterococci and their AR from the environment are scarce since enterococci are generally not considered important pathogens outside of hospitals and healthcare settings. A limited number of studies on environmental enterococci are mostly focused on water environments polluted by human inputs, such as wastewater, on species that are associated with human infections, such as *E. faecalis* and *E. faecium*, and on certain antimicrobial drugs, such as vancomycin [4,5,6,7]. Data concerning the presence of resistant enterococci in the aquatic environment is limited and still fewer data exists on the genetic content of waterborne antimicrobial resistant enterococci. A reservoir of resistant enterococci in the aquatic environment could potentially enhance the dissemination of resistance genes to other bacteria of the same genus and different genera in the environment via mobile genetic elements, such as conjugative plasmids [2]. Furthermore, enterococci are used as a sentinel organism for AR, whose resistance level may indicate the burden of AR in a population [8,9,10]. Monitoring resistant enterococci present in the environment and investigating a potential transfer of AR determinants to other bacteria in the same niche would enhance the understanding of AR from the one health perspective [11].

The significance of enterococci as clinically important pathogens is often associated with acquisition of AR via horizontal gene transfer using mobile genetic elements, especially plasmids in the strains. In our previous study, we demonstrated prevalence of enterococci that are resistant to antimicrobials commonly used in human and veterinary medicine from surface water of the Upper Oconee Watershed in GA, USA [12]. To further characterize AR in enterococci from freshwater environments, mechanisms conferring AR were examined. In addition, antimicrobial resistant enterococci were tested using a PCR-based plasmid typing system to identify and characterize plasmid replicons, and thus determine the plasmid contents of the antimicrobial resistant enterococci in the aquatic environment.

## 2. Materials and Methods

### 2.1. Enterococcus Isolates

Enterococci were isolated from surface water of the Upper Oconee Watershed, GA, USA, and their AR patterns were described in our previous study [12]. From the previous study, a total of 637 enterococci were isolated, of which, 93.1% (593/637) were resistant to at least one of the 16 antimicrobial drugs tested [12]. A total of 51 multidrug resistant (MDR; resistance ≥ 3 antimicrobial classes) *Enterococcus* isolates were used in the present study. They encompassed six different species, namely, *E. casseliflavus*, *E. faecalis*, *E. faecium*, *E. gallinarum*, *E. hirae*, and *E. mundtii*, exhibiting 18 different AR phenotypes with resistance up to six antimicrobial drugs (Table 1).

### 2.2. PCR

Isolates were tested for the presence of AR genes for the specific AR phenotype exhibited except for resistance to daptomycin and tigecycline, whose resistance mechanisms will be discussed elsewhere. Resistance determinants for chloramphenicol and penicillin were not determined as resistance to these antimicrobial drugs was seen in only one isolate each. Resistant isolates were tested by PCR for the presence of genes encoding resistance to aminoglycosides [*aac*(6′)-Ii, *aac*(6′)-Ie-*aph*(6″)-Ia, *aph*(6″)-Ib, *aph*(6″)-Ic, *aph*(6″)-Id, *aph*(3′)-IIIa, *ant*(3″)-Ia, *ant*(4′)-Ia, *ant*(6)-Ia, *ant*(9)-Ia], ciprofloxacin [*qnr_E. faecalis_*], macrolides [*erm*(A), *erm*(B) and *erm*(C)], lincosamides [*lnu*(A) and *lnu*(B)], Quinupristin/Dalfopristin (Q/D) [*vat*(A), *vat*(B), *vat*(C), *vat*(D) and *vat*(E)], and tetracycline [*tet*(K), *tet*(L), *tet*(M), *tet*(S), *tet*(O), *tet*(U)] as previously described (Supplementary Table S1) [13]. PCR assays were performed using whole-cell templates that were prepared by suspending a single bacterial colony in 200 µL of sterile deionized water. Amplified PCR products were separated by electrophoresis on a 1.5% agarose gel and visualized by staining with ethidium bromide.

Replicon families were determined as previously described using a PCR-based plasmid *rep*-typing system to detect 19 defined Gram-positive plasmid replicon families [14]: *rep*_1_ (prototype pIP501), *rep*_2_ (prototype pRE25), *rep*_3_ (prototype pAW63), *rep*_4_ (prototype pMBB1), *rep*_5_ (prototype pN315), *rep*_6_ (prototype pS86), *rep*_7_ (prototype pUSA02), *rep*_8_ (prototype pAM373), *rep*_9_ (prototype pCF10), *rep*_10_ (prototype pIM13), *rep*_11_ (prototype pEF1071), *rep*_12_ (pBMB67), *rep*_13_ (prototype pC194), *rep*_14_ (prototype pRI1), *rep*_15_ (prototype pUSA03), *rep*_16_ (prototype pSAS), *rep*_17_ (prototype pRUM), *rep*_18_ (prototype pEF418), *rep*_19_ (prototype pUB101), and the unique plasmid sequence of pMG1. PCR assays were performed using whole-cell templates, and amplified PCR products were separated by electrophoresis and visualized by staining with ethidium bromide as described above.

### 2.3. Statistical Methods

Pearson correlation coefficients were performed using Microsoft Excel. *p* values of ≤ 0.05 were considered significant.

## 3. Results

### 3.1. AR Genes

Twelve resistance genes were detected among the *Enterococcus* isolates (Table 2). Of the lincomycin-resistant isolates, 18.4% (9/49) contained *lnu*(B), while *lnu*(A) was not found in any of the isolates. Six enterococcal species were resistant to lincomycin, but *lnu*(B) was found in only two species, *E. faecium* and *E. hirae*. Of the 25 enterococcal isolates that were resistant to ciprofloxacin, three isolates contained *qnr_E. faecalis_*, all of which were *E. casseliflavus.* Only one out of the 12 Q/D-resistant isolates was positive for the presence of *vat*(E). Other Q/D resistance genes, *vat*(A), *vat*(B), *vat*(C), and *vat*(D), were not detected. Six of the seven enterococcal isolates that were resistant to macrolides i.e., erythromycin and tylosin, were positive for the presence of both *erm*(B) and *erm*(C), while *erm*(A) was not detected in any of the isolates.

Five tetracycline resistance genes (*tet*(K), *tet*(L), *tet*(M), *tet*(O) and *tet*(S)) were found in 93.9% (31/33) of the tetracycline-resistant isolates; *tet*(U) was not detected. *tet*(M) was most often detected (26/33; 78.8%) and was found across all five enterococcal species. Alternatively, *tet*(S) was only detected in *E. gallinarum*. *tet*(L) was found among four different enterococcal species, but the majority (5/8) was detected in *E. hirae*. Combinations of tetracycline resistance genes were not uncommon, with 12 tetracycline-resistant isolates carrying more than one resistance gene. Five different tetracycline gene combinations were exhibited: *tet*(K), *tet*(M); *tet*(L), *tet*(M); *tet*(M), *tet*(O); *tet*(K), *tet*(L), *tet*(M); *tet*(L), *tet*(M), *tet*(O) (Table 3). *tet*(L), *tet*(M) was the most common combination.

Of the four enterococcal isolates that were resistant to aminoglycosides, one isolate was resistant to kanamycin, two isolates were resistant to streptomycin, and one isolate was resistant to both kanamycin and streptomycin. *ant*(6)-Ia was observed in one out of the three streptomycin-resistant isolates, while *aph*(3′)-IIIa was observed in both isolates that were resistant to kanamycin. The isolate that was resistant to both kanamycin and streptomycin only carried one gene, *aph*(3′)-IIIa. 

Profiles of AR genes among the *Enterococcus* isolates are shown in Table 3. Not all the isolates with resistance phenotypes carried AR genes. Of the 51 MDR *Enterococcus* isolates that were tested for the presence of resistance genes, 15 of the isolates did not harbor any of the tested genes, most of which were *E. casseliflavus* (11/15; 73.3%). Of the 17 *E. casseliflavus* tested, only six isolates were positive for AR genes. However, all of the *E. faecalis* (*n* = 8) and *E. faecium* (*n* = 7) isolates were positive for at least one of the AR genes tested. AR gene patterns ranged from one resistance gene to five genes, with 44.4% (16/36) of isolates consisting of one resistance gene. The most common AR gene pattern was *tet*(M) (*n* = 7), followed by *tet*(S) (*n* = 4) and *lnu*(B), *tet*(L), *tet*(M) (*n* = 4).

### 3.2. Replicon Families

The presence of 19 *rep*-families was investigated among the 51 MDR *Enterococcus* isolates from surface water. Not all the *Enterococcus* isolates with AR phenotypes yielded amplicons for the *rep*-families, and the isolate with resistance to six antimicrobial classes was negative for all the *rep* genes tested. Two-thirds (35/51; 68.6%) of the enterococci were positive for at least one *rep*-family with up to 3 *rep*-families detected in four of the isolates (Table 4). Of the 19 *rep*-families tested, the MDR *Enterococcus* isolates represented 12 different *rep*-families. The *rep*-families detected and the number of *Enterococcus* isolates of different species that carried the *rep*-families are shown in Table 5. The most common *rep*-family among all the isolates was *rep*_11 (pEF1071)_ (*n* = 19), followed by *rep*_3 (pAW63)_ (*n* = 10). The majority of the *rep*_11 (pEF1071)_- and *rep*_3 (pAW63)_- positive isolates were *E. casseliflavus*, 78.9% (15/19) and 80.0% (8/10), respectively. The *rep*_9 (pCF10)_ family was only found in *E. faecalis.*

The number of different *rep*-families detected varied among *Enterococcus* isolates, with the greatest number of *rep*-families detected among *E. hirae* and the least number of *rep*-families among *E. faecium* (Table 5). Eight different *rep*-families were found among *E. hirae* (*rep*_3 (pAW63)_, *rep*_5 (pN315)_, *rep*_6 (pS86)_, *rep*_11 (pEF1071)_, *rep*_12 (pBMB67)_, *rep*_13 (pC194)_, *rep*_15 (pUSA03)_, *rep*_16 (pSAS)_), while only one *rep*-family was found among *E. faecium* (*rep*_2 (pRE25)_). Both *E. casseliflavus* (*rep*_2 (pRE25)_, *rep*_3 (pAW63)_, *rep*_5 (pN315)_, *rep*_11 (pEF1071)_, *rep*_12 (pBMB67)_, *rep*_16 (pSAS)_) and *E. faecalis* (*rep*_6 (pS86)_, *rep*_8 (pAM373)_, *rep*_9 (pCF10)_, *rep*_12 (pBMB67)_, *rep*_15 (pUSA03)_, *rep*_16 (pSAS)_) were positive for six *rep* amplicons, while four *rep* amplicons were detected in *E. gallinarum* (*rep*_4 (pMBB1)_, *rep*_5 (pN315)_, *rep*_11 (pEF1071)_, *rep*_15 (pUSA03)_). *E. mundtii*, for which there was only one isolate, was positive for three different *rep*-families, *rep*_3 (pAW63)_, *rep*_4 (pMBB1)_, and *rep*_11 (pEF1071)_.

Many of the *Enterococcus* isolates that belonged to the same species and had the same AR profiles had different *rep*-family profiles. For example, six *E. hirae* isolates with the same phenotypic resistance profile i.e., resistance to daptomycin, lincomycin, and tetracycline, had five different *rep*-family profiles i.e., *rep*_5 (pN315)_; *rep*_16 (pSAS)_; *rep*_6 (pS86)_, *rep*_12 (pBMB67)_; *rep*_3 (pAW63)_, *rep*_11 (pEF1071)_; *rep*_5 (pN315)_, *rep*_13 (pC194)_, *rep*_15 (pUSA03)_ (Table 1) suggesting that different plasmids can carry the same resistance genes to confer resistance to the same set of antimicrobial drugs. On the other hand, isolates with the same *rep*-family profiles exhibited different AR profiles in different species. For example, the same *rep*_3 (pAW63)_, *rep*_11 (pEF1071)_ profile was detected in six *E. casseliflavus* isolates that were resistant to either ciprofloxacin, lincomycin, and Q/D, or ciprofloxacin, lincomycin, and tigecycline, as well as in one *E. hirae* isolate that was resistant to daptomycin, lincomycin, and tetracycline (Table 1). This indicates that the same plasmids can acquire different sets of AR genes to exhibit different resistance profiles.

## 4. Discussion

In our previous study, most of the *Enterococcus* isolates from surface water were resistant to at least one antimicrobial drug with multiple resistant phenotypes identified in a total of 51 isolates [12]. For the current study, those isolates that were resistant to three or more antimicrobial classes were further tested to investigate the molecular mechanism of AR and the abundance and diversity of plasmids among enterococci from surface water. The study was undertaken to improve the understanding of AR in the environment from the perspective of the One Health approach.

Resistance to macrolides (erythromycin and tylosin) was attributed to *erm*(B) and *erm*(C), which is consistent with Mlynarczyk et al. who described *erm*(B) as the most prevalent gene conferring erythromycin resistance in enterococci [15]. Interestingly, *erm*(B) and *erm*(C) were detected together in all the isolates positive for macrolides resistance genes, an observation that was not commonly observed but previously reported in *E. faecalis* from cattle [16]. For lincomycin resistance, *lnu*(B) was the only gene detected, and this is consistent with the previous findings that while *lnu*(A) was originally described in *Staphylococcus*, *lnu*(B) has been described only in *Enterococcus* [17,18]. Only a small portion of the lincomycin-resistant isolates (18.4%) were positive for the gene, which was also reported in a previous study [13], suggesting that there may be mechanisms for lincomycin resistance among enterococci other than *lnu*(A) and *lnu*(B). Similarly, only one isolate out of the 12 Q/D-resistant isolates was positive for a Q/D resistance gene, *vat*(E), which encodes a streptogramin A acetyltransferase. Of all the Q/D resistance gene, *vat*(E) is the most frequent gene found in enterococci of different sources, including human, animals, and the environment [19,20,21].

A number of genes encoding tetracycline resistance were detected in several species, including *E. casseliflavus*, *E. faecalis*, *E. faecium*, *E. gallinarum*, and *E. hirae*. *tet*(M), which encodes for ribosomal protection protein, was the most frequently detected gene, followed by *tet*(L), which encodes for a tetracycline efflux, similar to the findings previously reported [4,13,22]. Tetracycline resistance is most often mediated by *tet*(M) in enterococci from humans, animals, food, and the environment [4,13,23,24,25]. *tet*(S) is a less commonly detected tetracycline gene in enterococci, but most of the studies that reported the presence of *tet*(S) were conducted on *E. faecalis* and *E. faecium* only [4,23,24]. This study detected five isolates with *tet*(S), and they were all *E. gallinarum*. Similarly, Cauwerts et al. detected *tet*(S) in six isolates from their study, all of which belonged to non*-faecalis*, non-*faecium* species, including *E. avium*, *E. casseliflavus*, and *E. gallinarum*, with three of the isolates identified as *E. gallinarum* [25]. This data, along with our finding, suggests that *tet*(S) is found more commonly in non-*faecalis* and non-*faecium* enterococcal species. Overall, most of the tetracycline-resistant enterococcal isolates (93.9%) carried *tet*(K), *tet*(L), *tet*(M), *tet*(O) and *tet*(S) alone or in combination, while *tet*(U) was not detected in any of the isolates. The resistance mechanism of *tet*(U), which is known to confer low-level resistance to tetracycline, is unknown, although a report by Caryl et al. questioned the role of *tet*(U) as a resistance determinant [26,27].

Kanamycin-resistant isolates were tested for eight different resistance genes, including *aac*(6′)-Ie-*aph*(6″)-Ia, which encodes a bifunctional enzyme that confers high-level resistance to all clinically important aminoglycosides except streptomycin, that is often detected in human and animal isolates [18,28]. However, both kanamycin-resistant isolates were positive for another frequently detected gene *aph*(3′)-IIIa, which confers high-level kanamycin resistance in enterococci [13,29,30,31]. Three streptomycin-resistant isolates were tested for the presence of the two commonly detected streptomycin resistance genes, but only one isolate carried *ant*(6)-Ia. The high prevalence of *ant*(6)-Ia was also observed in other studies, not only for human and animals but also for environmental water [4,13,30,32,33].

Three isolates, out of the 25 ciprofloxacin-resistant enterococcal isolates, were positive for *qnr_E. faecalis_.* This *qnr*-like gene, which was originally identified on the chromosome of *E. faecalis*, unlike the plasmid-borne fluoroquinolone resistance gene, *qnr*, found in *Enterobacteriaceae*, is known to contribute to fluoroquinolone resistance in *E. faecalis* [34]. However, none of the 25 ciprofloxacin-resistant isolates in this study were *E. faecalis*, and the three isolates carrying the *qnr_E. faecalis_* gene were identified as *E. casseliflavus*, suggesting that an investigation of *qnr*-like genes in enterococcal species other than *E. faecalis* is needed.

While AR genes found in human and animal isolates were observed in this study among environmental enterococci, indicating the presence of similar mechanisms conferring resistance among enterococci from different sources, many isolates were still negative for the AR genes tested. This indicates the lack of information on the resistance genes present in *Enterococcus*. Indeed, many *E. casseliflavus* isolates were negative for AR genes, with only six out of the 17 *E. casseliflavus* positive for any resistance genes tested, while all of the *E. faecalis* (*n* = 8) and *E. faecium* (*n* = 7) isolates were positive for at least one of the AR genes tested. This could indicate that the studies on the AR genes present in *Enterococcus* are mostly biased towards *E. faecium* and *E. faecalis*, and the understanding of the genetic mechanisms of AR in enterococcal species other than *E. faecium* and *E. faecalis* is still lacking. However, all of the *E. faecalis* and *E. faecium* isolates were resistant to tetracycline, and all but one isolate carried at least one of the tetracycline resistance genes. In contrast, half of the non-*faecalis*, non-*faecium* enterococcal isolates were susceptible to tetracycline. Most of the non-*faecalis*, non-*faecium* enterococcal isolates that were tetracycline-resistant were positive for at least one tetracycline resistance gene, while among those that were not resistant to tetracycline, only four isolates were positive for AR genes. This could indicate that the genetic mechanism for tetracycline resistance is well understood, but not for other drug resistances. A similar finding could be seen among enterococcal isolates from animal and food sources [13,35,36]. Although resistance mechanisms for tetracycline and erythromycin were well understood and present among most of the enterococcal isolates across all species, AR genes could not be identified for many other antimicrobial drugs [13,35,36]. The genetic mechanisms of AR in non-*faecalis* and non-*faecium* enterococcal species seem to be quite different from those of *E. faecalis* and *E. faecium*, and the understanding of AR mechanisms needs to be improved for different species.

A third of the MDR *Enterococcus* isolates did not carry any plasmids of 19 replicon families. This is similar to the previous result by Jensen et al., in which 32% of *E. faecalis* and 33% of *E. faecium* from their set of human and animal isolates did not harbor any plasmids of 19 *rep*-families [14]. However, most of the isolates without *rep* genes were from pigs, and only three isolates, out of the 26 isolates without *rep* genes, were human clinical isolates [14]. This is similar to other studies that conducted plasmid replicon typing PCR on human isolates. Rosvoll et al. and Wardal et al. detected only 4% (4/99) of *E. faecium* and 1.3% (2/152) of *E. faecalis*, respectively, without any *rep* genes [37,38]. This indicates the presence of plasmids of novel *rep*-families that are yet to be defined or unique sequences that were not tested, or the presence of other mobile genetic elements that may contain AR genes in non-human enterococcal isolates.

Twelve *rep*-families were detected in this study, indicating the presence of 12 different plasmids among the 51 MDR *Enterococcus* isolates from surface water, suggesting the diversity of plasmids among enterococci with the indication of different mechanisms for AR gene transfer. This is a greater variety of *rep*-families compared to other studies that detected between five and nine *rep*-families using the same PCR-based *rep*-typing method [4,14,31,37,38,39,40]. Some of the *rep*-families that were previously not detected among the enterococci were detected in the current study, including *rep*_3 (pAW63)_, *rep*_5 (pN315)_, *rep*_12 (pBMB67)_, *rep*_15 (pUSA03)_, and *rep*_16 (pSAS)_. The species in which *rep*_3 (pAW63)_ and *rep*_5 (pN315)_ plasmids were detected were non-*faecalis*, non-*faecium* enterococcal species, i.e., *E. casseliflavus*, *E. gallinarum*, *E. hirae*, and *E. mundtii*. This result demonstrates diverse plasmid contents among different species of *Enterococcus*. The *rep*-families that were commonly found in *E. faecalis* and *E. faecium* isolates from previously conducted studies were not detected in this study among the same species, including *rep*_1 (pIP501)_, *rep*_7 (pUSA02)_, *rep*_14 (pRI)_, *rep*_17 (pRUM)_, and *rep*_18 (pEF418)_ [4,14,31,37,38,39,40]. This may suggest a loss of plasmids, or a change in plasmid content, in *E. faecalis* and *E. faecium* in the environment with less selective pressure of antimicrobials. Moreover, the results of this study revealed the presence of multiple *rep* genes in half of the isolates with positive *rep* genes, indicating a high abundance of plasmids among enterococci of environmental origin. However, the average number of *rep* genes present per isolate was lower than the number of *rep* genes detected in isolates of clinical origin [37,38,40], underscoring the loss of plasmids in the environment with lower antimicrobial levels.

The most common *rep*-families identified among *E. faecalis* in the previous studies from their sets of human and animal isolates were *rep*_9 (pCF10)_ [14,38,39]. Similarly, the current study consisting of environmental isolates found that *rep*_9 (pCF10)_ was the most frequently detected *rep*-family among *E. faecalis*. The *rep*-families that were detected among *E. faecalis* in the current study were commonly detected in other studies as well i.e., *rep*_2 (pRE25)_, *rep*_6 (pS86)_, *rep*_8 (pAM373)_, and *rep*_9 (pCF10)_, while *rep*_12 (pBMB67)_, *rep*_15 (pUSA03)_, and *rep*_16 (pSAS)_ had not been identified in other studies [14,38,39]. This suggests a variability in plasmid content among the isolates of the same species from different sources. Three isolates were positive for *rep*_8 (pAM373)_ and/or *rep*_9 (pCF10)_, and they were all *E. faecalis*. This was not surprising as pAM373 of *rep*_8 (pAM373)_ as well as pAD1 and pCF10 of *rep*_9 (pCF10)_ are pheromone-responsive plasmids, which are known to exist primarily in *E. faecalis* as most of the pheromone-responsive plasmids have been described in this species [41]. In contrast to the notion that pheromone-responsive plasmids enhance the acquisition of resistance, the isolates that harbored *rep*_8 (pAM373)_ and *rep*_9 (pCF10)_ plasmids in this study did not carry multiple AR genes and only harbored *tet*(M).

Only one isolate out of the seven *E. faecium* isolates tested, was positive for a *rep* gene, which is a much lower frequency compared to those of the previous studies with detection rates of *rep* genes between 67% and 100% of the total *E. faecium* isolates [4,14,31,37,40]. In contrast to the previous studies that indicate *E. faecium* of the clinical origin as the reservoir of plasmids of various *rep*-types, the current study only detected one *rep* gene in one isolate among MDR *E. faecium* isolates, and this might suggest the variation of plasmid content in enterococci of various source type and a loss of AR plasmids in less-selective environments. Enterococci may lose their AR plasmids in the environment where antimicrobials are less intensively used as they may be a burden for the host [2]. The only *rep*-family detected among *E. faecium* in this study was *rep*_2 (pRE25)_, which was the most prevalent *rep*-family in *E. faecium* previously identified [4,14,31,37,40]. pRE25, which is a member of *rep*_2_ family, is a conjugative multi-resistance plasmid; therefore, the presence of *rep*_2 (pRE25)_ plasmid in the MDR *E. faecium* recovered from the aquatic environment was not surprising [42]. The only other species that was positive for *rep*_2 (pRE25)_ was *E. casseliflavus*, which is the first instance that this plasmid has been reported in *E. casseliflavus*. An environmental *E. hirae* isolated from marine sediment was reported to carry pRE25, and the isolate was resistant to erythromycin, similar to the *rep*_2 (pRE25)_-positive *E. casseliflavus* isolate from this study [43]. The same group also reported an *E. faecium* isolate positive for pRE25 in marine sediment [44]. These results suggest the frequent presence of this conjugative multi-resistance plasmid in the environment capable of transfer, along with AR genes, to other bacteria [43,44].

The most dominant *rep*-family among all the isolates in this study was *rep*_11 (pEF1071)_ (*n* = 19), followed by *rep*_3 (pAW63)_ (*n* = 10). Most of the *rep*_11 (pEF1071)_ and *rep*_3 (pAW63)_ were detected in *E. casseliflavus*, 78.9% and 80% respectively, with a significant positive association (*p* < 0.05) between the plasmids belonging to *rep*_11 (pEF1071)_ and *rep*_3 (pAW63)_ with *E. casseliflavus*. Even though *rep*_11 (pEF1071)_ had been detected in *E. faecium* isolates in several studies, this was the first time *rep*_11 (pEF1071)_ was reported in *E. casseliflavus* [31,37,40]. pEF1071 is a member of *rep*_11_ family, and enterocins 1071A and 1071B are known to be encoded on pEF1071 [45]. Since enterocins exhibit antimicrobial activity and the producer bearing the plasmid would have a survival advantage over other closely related species present in the same ecological niche [45], it is possible that the presence of pEF1071 enabled *E. casseliflavus* to be the most frequently identified species and also the most common species with MDR phenotypes found in the surface water [12].

To date, most of studies on the occurrence and distribution of AR genes and plasmids in *Enterococcus* have focused on human or animal isolates, while data on the distribution of AR genes in *Enterococcus* recovered from the environment are relatively limited. Moreover, no study has attempted to classify plasmids present in environmental enterococci, especially those including non-*faecalis*, non-*faecium* enterococcal species. To the best of our knowledge, this is one of the first investigations, if not the first, of molecular characterization of MDR *Enterococcus* from surface water that encompasses a significant number of enterococcal species.

## 5. Conclusions

The mechanisms conferring resistance were determined among the MDR enterococci, but the observation that AR mechanisms could not be determined for many of the resistant isolates indicates that diverse AR mechanisms exist among environmental enterococci, and the understanding of AR mechanisms for enterococci is limited. Furthermore, the plasmid contents of the enterococci in the aquatic environment appears to be quite different from those in hospital settings and livestock. Diverse plasmids were identified among enterococci isolated from surface water, which indicates that environmental enterococci may carry multiple plasmids of different *rep*-families through which AR genes can be transferred. Moreover, pheromone-responsive plasmids, which could be an effective vector for AR dissemination, were present in surface water. This study indicates that surface water may be a suitable environment for the transfer of AR genes via horizontal gene transfer through plasmids harbored by enterococcal isolates present in the aquatic environment. This may result in the transfer of AR determinants to human strains and contribute to human infections through exposure to contaminated water.

## Figures and Tables

**Table 1:** Masterfile of multidrug resistant (MDR) enterococci from surface water with their antimicrobial resistance (AR) gene and *rep*-family profiles.

Isolate ID	Species	AR Phenotype ^1^	AR Gene	Plasmid	
				*Rep*-Family	Prototype
2 Ent	*E. faecium*	CipLinTet	*tet*(M), *tet*(O)		
2 mE	*E. faecium*	CipLinTet	*tet*(M), *tet*(O)		
3 mE	*E. casseliflavus*	CipLinSyn		3, 11	pAW63, pEF1071
7 mE	*E. casseliflavus*	CipLinSyn		11	pEF1071
23 mE	*E. gallinarum*	CipLinTet	*tet*(S)	11	pEF1071
24 Ent	*E. faecium*	CipDapTet	*tet*(M)		
24 mE	*E. faecium*	CipDapTet	*tet*(M)		
25 mE	*E. faecium*	CipEryLinTetTyl	*lnu*(B), *tet*(L), *tet*(M), *erm*(B), *erm*(C)	2	pRE25
25 Ent	*E. faecium*	LinStrTet	*ant*(6)-Ia		
26 mE	*E. casseliflavus*	CipLinSyn		3, 11, 16	pAW63, pEF1071, pSAS16
27 Ent	*E. hirae*	DapLinTet	*lnu*(B), *tet*(L), *tet*(M)	5, 13, 15	pSAS, pC194, pUSA03
72 mE	*E. casseliflavus*	KanLinStrTetTyl	*tet*(M), *erm*(B), *erm*(C), *aph*(3′)-IIIa	3, 11, 12	pAW63, pEF1071, pBMB67
97 mE	*E. casseliflavus*	CipLinSyn		3, 11	pAW63, pEF1071
101 mE	*E. casseliflavus*	CipLinTet	*tet*(M), *qnr_E. faecalis_*	11	pEF1071
118 mE	*E. faecalis*	ChlDapEryLinTetTyl	*tet*(M), *erm*(B), *erm*(C)		
131c- mE	*E. hirae*	DapLinTet	*lnu*(B), *tet*(L), *tet*(M)	5	pSAS
134a- mE	*E. gallinarum*	EryLinTetTyl	*tet*(S), *erm*(B), *erm*(C)		
134c- CHR	*E. gallinarum*	EryLinSynTetTyl	*tet*(S)		
137a- Ent	*E. faecalis*	LinPenTet	*tet*(M)	9	pCF10
139a- Ent	*E. gallinarum*	CipLinTet	*tet*(K), *tet*(M)	11, 15	pEF1071, pUSA03
139b- Ent	*E. gallinarum*	CipLinTet	*tet*(M)		
157a- mE	*E. hirae*	DapLinTet	*lnu*(B), *tet*(L), *tet*(M)	16	pSAS16
160a- Ent	*E. faecalis*	EryLinTetTyl	*tet*(M), *erm*(B), *erm*(C)		
160b- mE	*E. faecalis*	EryLinTetTyl	*tet*(M), *erm*(B), *erm*(C)	16	pSAS16
163a- mE	*E. hirae*	DapLinTet	*lnu*(B), *tet*(L), *tet*(M), *tet*(O)	5	pSAS
164 Ent	*E. casseliflavus*	CipLinTig		11	pEF1071
168 Ent	*E. casseliflavus*	LinSynTig		11	pEF1071
169 Ent	*E. casseliflavus*	CipLinTig	*qnr_E. faecalis_*	3, 11	pAW63, pEF1071
170 Ent	*E. gallinarum*	LinSynTig			
173 Ent	*E. casseliflavus*	CipLinTig		3, 11	pAW63, pEF1071
177 Ent	*E. gallinarum*	LinSynTig			
182 Ent	*E. casseliflavus*	LinSynTig		11	pEF1071
184 Ent	*E. casseliflavus*	CipLinTig		3, 11	pAW63, pEF1071
193 Ent	*E. casseliflavus*	CipLinTig		3, 11	pAW63, pEF1071
206 Ent	*E. casseliflavus*	LinSynTig	*vat*(E)	11	pEF1071
222 Ent	*E. casseliflavus*	CipLinTetTig			
251 Ent	*E. faecalis*	LinStrTet	*tet*(M)	8, 9	pAM373, pCF10
253 Ent	*E. hirae*	DapLinTet	*lnu*(B), *tet*(L), *tet*(M)	6, 12	pS86, pBMB67
262 Ent	*E. faecium*	CipLinTet	*lnu*(B), *tet*(M)		
314 Ent	*E. gallinarum*	CipLinTet	*tet*(S)		
317 Ent	*E. hirae*	DapLinTet	*lnu*(B), *tet*(M)	3, 11	pAW63, pEF1071
339 Ent	*E. casseliflavus*	EryLinTetTyl	*tet*(L), *tet*(M), *erm*(B), *erm*(C)	2, 5	pRE25, pSAS
379 Ent	*E. mundtii*	DapLinTig		3, 4, 11	pAW63, pMBB1, pEF1071
384 Ent	*E. casseliflavus*	CipLinSynTig	*qnr_E. faecalis_*	11	pEF1071
394 Ent	*E. hirae*	DapLinTig	*lnu*(B)	6	pS86
396 Ent	*E. gallinarum*	CipLinTetTig	*tet*(S)	5	pSAS
407 Ent	*E. faecalis*	KanLinTet	*tet*(K), *tet*(L), *tet*(M), *aph*(3′)-IIIa	6, 12	pS86, pBMB67
420 Ent	*E. hirae*	CipLinSyn			
421 Ent	*E. gallinarum*	CipLinTetTig	*tet*(M)	4	pMBB1
423 Ent	*E. faecalis*	LinTetTig	*tet*(M)	9, 15	pCF10, pUSA03
436 Ent	*E. faecalis*	LinTetTig	*tet*(K), *tet*(M)	15	pUSA03

^1^ Antimicrobials: Chloramphenicol (Chl), Ciprofloxacin (Cip), Daptomycin (Dap), Erythromycin (Ery), Kanamycin (Kan), Lincomycin (Lin), Penicillin (Pen), Synercid (Q/D) (Syn), Streptomycin (Str), Tetracycline (Tet), Tigecycline (Tig), and Tylosin (Tyl).

**Table 2:** AR genes detected among MDR enterococci from surface water.

AR Phenotype (No. of Isolates)	AR Gene	No. of Gene (%)	Species (No. of Isolates)
Lincomycin (*n* = 49)	*lnu*(B)	9 (18.4)	*E. faecium* (2)
			*E. hirae* (7)
Tetracycline (*n* = 33)	*tet*(K)	3 (9.1)	*E. faecalis* (2)
			*E. gallinarum* (1)
	*tet*(L)	8 (24.2)	*E. casseliflavus* (1)
			*E. faecalis* (1)
			*E. faecium* (1)
			*E. hirae* (5)
	*tet*(M)	26 (78.8)	*E. casseliflavus* (3)
			*E. faecalis* (8)
			*E. faecium* (6)
			*E. gallinarum* (3)
			*E. hirae* (6)
	*tet*(O)	3 (9.1)	*E. faecium* (2)
			*E. hirae* (1)
	*tet*(S)	5 (15.2)	*E. gallinarum* (5)
Ciprofloxacin (*n* = 25)	*qnr_E. faecalis_*	3 (12.0)	*E. casseliflavus* (3)
Q/D (*n* = 12)	*vat*(E)	1 (8.3)	*E. casseliflavus* (1)
Macrolides (tylosin, erythromycin) (*n* = 7)	*erm*(B)	6 (85.7)	*E. casseliflavus* (1)
			*E. faecalis* (3)
			*E. faecium* (1)
			*E. gallinarum* (1)
	*erm*(C)	6 (85.7)	*E. casseliflavus* (1)
			*E. faecalis* (3)
			*E. faecium* (1)
			*E. gallinarum* (1)
Streptomycin (*n* = 3)	*ant*(6)-Ia	1 (33.3)	*E. faecium* (1)
Kanamycin (*n* = 2)	*aph*(3′)-IIIa	2 (100.0)	*E. casseliflavus* (1)
			*E. faecalis* (1)

**Table 3:** Profiles of AR genes detected among enterococci isolated from surface water.

AR Gene Profile	No. of Isolates	Species (No. of Isolates)
none	15	*E. casseliflavus* (11), *E. gallinarum* (2), *E. hirae* (1), *E. mundtii* (1)
*ant*(6)-Ia	1	*E. faecium* (1)
*lnu*(B)	1	*E. hirae* (1)
*qnr_E. faecalis_*	2	*E. casseliflavus* (2)
*tet*(M)	7	*E. faecium* (2), *E. faecalis* (3), *E. gallinarum* (2)
*tet*(S)	4	*E. gallinarum* (4)
*vat*(E)	1	*E. casseliflavus* (1)
*lnu*(B), *tet*(M)	2	*E. faecium* (1), *E. hirae* (1)
*tet*(K), *tet*(M)	2	*E. gallinarum* (1)*, E. faecalis* (1)
*tet*(M), *qnr_E. faecalis_*	1	*E. casseliflavus* (1)
*tet*(M), *tet*(O)	2	*E. faecium* (2)
*lnu*(B), *tet*(L), *tet*(M)	4	*E. hirae* (4)
*tet*(M), *erm*(B), *erm*(C)	3	*E. faecalis* (3)
*tet*(S), *erm*(B), *erm*(C)	1	*E. gallinarum* (1)
*lnu*(B), *tet*(L), *tet*(M), *tet*(O)	1	*E. hirae* (1)
*tet*(K), *tet*(L), *tet*(M), *aph*(3′)-IIIa	1	*E. faecalis* (1)
*tet*(L), *tet*(M), *erm*(B), *erm*(C)	1	*E. casseliflavus* (1)
*tet*(M), *erm*(B), *erm*(C), *aph*(3′)-IIIa	1	*E. casseliflavus* (1)
*lnu*(B), *tet*(L), *tet*(M), *erm*(B), *erm*(C)	1	*E. faecium* (1)

**Table 4:** Profiles of *rep*-families detected among MDR enterococci from surface water.

No. of *Rep*-Families	Plasmid		No. of Isolates	Species (No. of Isolates)
	*Rep*-Family	Prototype		
0			16	*E. casseliflavus* (1), *E. faecalis* (2), *E. faecium* (6), *E. gallinarum* (6), *E. hirae* (1)
1	2	pRE25	1	*E. faecium* (1)
	5	pSAS	3	*E. gallinarum* (1), *E. hirae* (2)
	4	pMBB1	1	*E. gallinarum* (1)
	6	pS86	1	*E. hirae* (1)
	9	pCF10	1	*E. faecalis* (1)
	11	pEF1071	8	*E. casseliflavus* (7), *E. gallinarum* (1)
	15	USA03	1	*E. faecalis* (1)
	16	pSAS16	2	*E. faecalis* (1), *E. hirae* (1)
2	3, 11	pAW63, pEF1071	7	*E. casseliflavus* (6), *E. hirae* (1)
	2, 5	pRE25, pSAS	1	*E. casseliflavus* (1)
	8, 9	pAM373, pCF10	1	*E. faecalis* (1)
	9, 15	pCF10, pUSA03	1	*E. faecalis* (1)
	6, 12	pS86, pBMB67	2	*E. faecalis* (1), *E. hirae* (1)
	11, 15	pEF1071, pUSA03	1	*E. gallinarum* (1)
3	3, 4, 11	pAW63, pMBB1, pEF1071	1	*E. mundtii* (1)
	3, 11, 16	pAW63, pEF1071, pSAS16	1	*E. casseliflavus* (1)
	3, 11, 12	pAW63, pEF1071, pBMB67	1	*E. casseliflavus* (1)
	5, 13, 15	pSAS, pC194, pUSA03	1	*E. hirae* (1)

**Table 5:** Frequency of *rep*-families detected among MDR enterococci from surface water.

Plasmid		No. of Isolates	Species (No. of Isolates)
*Rep*-Family	Prototype		
2	pRE25	2	*E. casseliflavus* (1), *E. faecium* (1)
3	pAW63	10	*E. casseliflavus* (8), *E. hirae* (1), *E. mundtii* (1)
4	pMBB1	2	*E. gallinarum* (1), *E. mundtii (1)*
5	pSAS	5	*E. casseliflavus* (1), *E. gallinarum* (1), *E. hirae* (3)
6	pS86	3	*E. faecalis* (1), *E. hirae* (2)
8	pAM373	1	*E. faecalis* (1)
9	pCF10	3	*E. faecalis* (3)
11	pEF1071	19	*E. casseliflavus* (15), *E. gallinarum* (2), *E. hirae* (1), *E. mundtii* (1)
12	pBMB67	3	*E. casseliflavus* (1), *E. faecalis* (1), *E. hirae* (1)
13	pC194	1	*E. hirae* (1)
15	pUSA03	4	*E. faecalis* (2), *E. gallinarum* (1), *E. hirae* (1)
16	pSAS16	3	*E. casseliflavus* (1), *E. faecalis* (1), *E. hirae* (1)

## Supplementary Materials

The following table is available online at https://www.mdpi.com/2076-2607/8/9/1338/s1, Table S1: Primers used for the identification of AR genes in MDR enterococci from surface water.

## References

[1] Tannock G.W., Cook G., Gilmore M., Clewell D., Courvalin P., Dunny G., Murray B., Rice L. (2002). Enterococci as members of the intestinal microflora of humans. The Enterococci: Pathogenesis, Molecular Biology, and Antibiotic Resistance.

[2] Werner G., Coque T.M., Franz C.M., Grohmann E., Hegstad K., Jensen L., van Schaik W., Weaver K. (2013). Antibiotic resistant enterococci-tales of a drug resistance gene trafficker. Int. J. Med. Microbiol..

[3] Arias C.A., Murray B.E. (2012). The rise of the *Enterococcus*: Beyond vancomycin resistance. Nat. Rev. Microbiol..

[4] Sadowy E., Luczkiewicz A. (2014). Drug-resistant and hospital-associated *Enterococcus faecium* from wastewater, riverine estuary and anthropogenically impacted marine catchment basin. BMC Microbiol..

[5] Nishiyama M., Iguchi A., Suzuki Y. (2015). Identification of *Enterococcus faecium* and *Enterococcus faecalis* as *van*C-type Vancomycin-Resistant Enterococci (VRE) from sewage and river water in the provincial city of Miyazaki, Japan. J. Environ. Sci. Health A Toxic Hazard. Subst. Environ. Eng..

[6] Sanderson H., Ortega-Polo R., McDermott K., Hall G., Zaheer R., Brown R.S., Majury A., McAllister T.A., Liss S.N. (2019). Quantification and multidrug resistance profiles of vancomycin-resistant enterococci isolated from two wastewater treatment plants in the same municipality. Microorganisms.

[7] Young S., Nayak B., Sun S., Badgley B.D., Rohr J.R., Harwood V.J. (2016). Vancomycin-resistant enterococci and bacterial community structure following a sewage spill into an aquatic environment. Appl. Environ. Microbiol..

[8] Bager F. (2000). DANMAP: Monitoring antimicrobial resistance in Denmark. Int. J. Antimicrob. Agents.

[9] FDA (2013). National Antimicrobial Resistance Monitoring System-Enteric Bacteria (NARMS): 2011 Retail Meat Annual Report.

[10] WHO (2013). Integrated Surveillance of Antimicrobial Resistance: Guidance from a WHO Advisory Group.

[11] Robinson T.P., Bu D.P., Carrique-Mas J., Fevre E.M., Gilbert M., Grace D., Hay S.I., Jiwakanon J., Kakkar M., Kariuki S. (2016). Antibiotic resistance is the quintessential One Health issue. Trans. R. Soc. Trop. Med. Hyg..

[12] Cho S., Hiott L.M., McDonald J.M., Barrett J.B., McMillan E.A., House S.L., Adams E.S., Frye J.G., Jackson C.R. (2019). Diversity and antimicrobial resistance of *Enterococcus* from the Upper Oconee Watershed, Georgia. J. Appl. Microbiol..

[13] Jackson C.R., Fedorka-Cray P.J., Davis J.A., Barrett J.B., Brousse J.H., Gustafson J., Kucher M. (2010). Mechanisms of antimicrobial resistance and genetic relatedness among enterococci isolated from dogs and cats in the United States. J. Appl. Microbiol..

[14] Jensen L.B., Garcia-Migura L., Valenzuela A.J.S., Lohr M., Hasman H., Aarestrup F.M. (2010). A classification system for plasmids from enterococci and other Gram-positive bacteria. J. Microbiol. Methods.

[15] Mlynarczyk B., Mlynarczyk A., Kmera-Muszynska M., Majewski S., Mlynarczyk G. (2010). Mechanisms of resistance to antimicrobial drugs in pathogenic Gram-positive cocci. Mini-Rev. Med. Chem..

[16] Yang F., Zhang S., Shang X., Wang X., Yan Z., Li H., Li J. (2019). Short communication: Antimicrobial resistance and virulence genes of *Enterococcus faecalis* isolated from subclinical bovine mastitis cases in China. J. Dairy Sci..

[17] Brisson-Noël A., Courvalin P. (1986). Nucleotide sequence of gene *linA* encoding resistance to lincosamides in *Staphylococcus haemolyticus*. Gene.

[18] Frye J.G., Jackson C.R. (2013). Genetic mechanisms of antimicrobial resistance identified in *Salmonella enterica*, *Escherichia coli*, and *Enterococcus* spp. isolated from U.S. food animals. Front. Microbiol..

[19] Soltani M., Beighton D., Philpott-Howard J., Woodford N. (2000). Mechanisms of resistance to quinupristin-dalfopristin among isolates of *Enterococcus faecium* from animals, raw meat, and hospital patients in Western Europe. Antimicrob. Agents Chemother..

[20] Werner G., Klare I., Heier H., Hinz K.H., Böhme G., Wendt M., Witte W. (2000). Quinupristin-dalfopristin–resistant enterococci of the *satA* (*vatD*) and *satG* (*vatE*) genotypes from different ecological origins in Germany. Microb. Drug Resist..

[21] Donabedian S.M., Perri M.B., Vager D., Hershberger E., Malani P., Simjee S., Chow J., Vergis E.N., Muder R.R., Gay K. (2006). Quinupristin-dalfopristin resistance in *Enterococcus faecium* isolates from humans, farm animals, and grocery store meat in the United States. J. Clin. Microbiol..

[22] Roberts M.C. (2005). Update on acquired tetracycline resistance genes. FEMS Microbiol. Lett..

[23] Aarestrup F.M., Agerso Y., Gerner-Smidt P., Madsen M., Jensen L.B. (2000). Comparison of antimicrobial resistance phenotypes and resistance genes in *Enterococcus faecalis* and *Enterococcus faecium* from humans in the community, broilers, and pigs in Denmark. Diagn. Microbiol. Infect. Dis..

[24] Huys G., D’Haene K., Collard J.M., Swings J. (2004). Prevalence and molecular characterization of tetracycline resistance in *Enterococcus* isolates from food. Appl. Environ. Microbiol..

[25] Cauwerts K., Decostere A., De Graef E.M., Haesebrouck F., Pasmans F. (2007). High prevalence of tetracycline resistance in *Enterococcus* isolates from broilers carrying the *erm*(B) gene. Avian Pathol..

[26] Ridenhour M.B., Fletcher H.M., Mortensen J.E., Daneo-Moore L. (1996). A novel tetracycline-resistant determinant, *tet*(U), is encoded on the plasmid pKQ10 in *Enterococcus faecium*. Plasmid.

[27] Caryl J.A., Cox G., Trimble S., O’Neill A.J. (2012). “*tet*(U)” is not a tetracycline resistance determinant. Antimicrob. Agents Chemother..

[28] Torres C., Alonso C.A., Ruiz-Ripa L., León-Sampedro R., Del Campo R., Coque T.M. (2018). Antimicrobial Resistance in *Enterococcus* spp. of animal origin. Microbiol. Spectr..

[29] Chow J.W. (2000). Aminoglycoside resistance in enterococci. Clin. Infect..

[30] Jackson C.R., Fedorka-Cray P.J., Barrett J.B., Ladely S.R. (2004). Effects of tylosin use on erythromycin resistance in enterococci isolated from swine. Appl. Environ. Microbiol..

[31] Tremblay C.L., Charlebois A., Masson L., Archambault M. (2013). Characterization of hospital-associated lineages of ampicillin-resistant *Enterococcus faecium* from clinical cases in dogs and humans. Front. Microbiol..

[32] Veljović K., Popović N., Vidojević A.T., Tolinački M., Mihajlović S., Jovčić B., Kojić M. (2015). Environmental waters as a source of antibiotic-resistant *Enterococcus* species in Belgrade, Serbia. Environ. Monit. Assess..

[33] Zaheer R., Cook S.R., Barbieri R., Goji N., Cameron A., Petkau A., Polo R.O., Tymensen L., Stamm C., Song J. (2020). Surveillance of *Enterococcus* spp. reveals distinct species and antimicrobial resistance diversity across a One-Health continuum. Sci. Rep..

[34] Arsène S., Leclercq R. (2007). Role of a *qnr*-like gene in the intrinsic resistance of *Enterococcus faecalis* to fluoroquinolones. Antimicrob. Agents Chemother..

[35] Cui P., Feng L., Zhang L., He J., An T., Fu X., Li C., Zhao X., Zhai Y., Li H. (2020). Antimicrobial resistance, virulence genes, and biofilm formation capacity among *Enterococcus* species from yaks in Aba Tibetan Autonomous Prefecture, China. Front. Microbiol..

[36] Igbinosa E.O., Beshiru A. (2019). Antimicrobial resistance, virulence determinants, and biofilm formation of *Enterococcus* species from ready-to-eat seafood. Front. Microbiol..

[37] Rosvoll T.C., Lindstad B.L., Lunde T.M., Hegstad K., Aasnaes B., Hammerum A.M., Lester C.H., Simonsen G.S., Sundsfjord A., Pedersen T. (2012). Increased high-level gentamicin resistance in invasive *Enterococcus faecium* is associated with *aac(6′)Ie-aph(6″)Ia*-encoding transferable megaplasmids hosted by major hospital-adapted lineages. FEMS Immunol. Med. Microbiol..

[38] Wardal E., Gawryszewska I., Hryniewicz W., Sadowy E. (2013). Abundance and diversity of plasmid-associated genes among clinical isolates of *Enterococcus faecalis*. Plasmid.

[39] Song X., Sun J., Mikalsen T., Roberts A.P., Sundsfjord A. (2013). Characterisation of the plasmidome within *Enterococcus faecalis* isolated from marginal periodontitis patients in Norway. PLoS ONE.

[40] Wardal E., Kuch A., Gawryszewska I., Żabicka D., Hryniewicz W., Sadowy E. (2017). Diversity of plasmids and Tn*1546*-type transposons among VanA *Enterococcus faecium* in Poland. Eur. J. Clin. Microbiol. Infect. Dis..

[41] Clewell D.B., Dunny G.M., Gilmore M., Clewell D., Courvalin P., Dunny G., Murray B., Rice L. (2002). Conjugation and genetic exchange in enterococci. The Enterococci: Pathogenesis, Molecular Biology, and Antibiotic Resistance.

[42] Schwarz F.V., Perreten V., Teuber M. (2001). Sequence of the 50-kb conjugative multiresistance plasmid pRE25 from *Enterococcus faecalis* RE25. Plasmid.

[43] Pasquaroli S., Di Cesare A., Vignaroli C., Conti G., Citterio B., Biavasco F. (2014). Erythromycin- and copper-resistant *Enterococcus hirae* from marine sediment and co-transfer of *erm*(B) and *tcr*B to human *Enterococcus faecalis*. Diagn. Microbiol. Infect. Dis..

[44] Morroni G., Di Cesare A., Di Sante L., Brenciani A., Vignaroli C., Pasquaroli S., Giovanetti E., Sabatino R., Rossi L., Magnani M. (2016). *Enterococcus faecium* ST17 from coastal marine sediment carrying transferable multidrug resistance plasmids. Microb. Drug. Resist..

[45] Balla E., Dicks L.M.T. (2005). Molecular analysis of the gene cluster involved in the production and secretion of enterocins 1071A and 1071B and of the genes responsible for the replication and transfer of plasmid pEF1071. Int. J. Food Microbiol..

